# Positive renal familial history in IgA nephropathy is associated with worse renal outcomes: a single-center longitudinal study

**DOI:** 10.1186/s12882-021-02425-8

**Published:** 2021-06-19

**Authors:** Yoshinori Sato, Hiroyasu Tsukaguchi, Koichiro Higasa, Naoto Kawata, Kiyoko Inui, Tran Nguyen Truc Linh, Tran Thuy Huong Quynh, Inoue Yoshihiko, Fumihiko Koiwa, Ashio Yoshimura

**Affiliations:** 1 Department of Internal Medicine, Division of Nephrology, Showa University School of Medicine, Fujigaoka Hospital, 1-30 Fujigaoka, Aobaku, Yokohama, Kanagawa, 227-8501 Japan; 2 2nd Department of Internal Medicine, Kansai Medical University, Hirakata, Osaka 573-1010, Japan; 3 Department of Genome Analysis, Institute of Biomedical Science, Kansai Medical University, Hirakata, Osaka 573-1010, Japan; 4 Shinyokohama-Daiichi Clinic, Yokohama, Kanagawa, Japan

**Keywords:** IgA nephropathy, End-stage renal disease, Familial history, Genetic factor, Proteinuria

## Abstract

**Background:**

IgA nephropathy (IgAN) is the most common primary glomerulonephritis worldwide. Although most IgAN cases are sporadic, few show a familial aggregation. However, the prevalence and prognosis of IgAN individuals with positive familial history (FH) of renal disorders remains uncertain. To address these issues, we conducted a longitudinal observational study on a single-institution cohort of patients with biopsy-proven IgAN.

**Methods:**

A total of 467 IgAN patients who underwent renal biopsy during 1994 to 2019 were ascertained to have positive- or negative-FH by history taking and were followed for an average of 8.9 years. We compared the clinical and pathological features of the two subgroups. The primary outcome, a composite of a hard endpoint (end-stage renal disease [ESRD]) and surrogate endpoint (a 50% or more reduction in the estimated glomerular filtration rate [eGFR] from baseline), was evaluated. To estimate the risk for progression to ESRD, a Cox proportional hazards analysis was performed for a subset of patients who underwent follow-up for > 2 years and had an eGFR > 30 mL/min/1.73 m^2^ at baseline (*n* = 389; observation, 8.7 years).

**Results:**

Positive-FH subtype accounted for 11.6% (*n* = 54) of all IgAN patients. At baseline, there were no significant differences between the positive- and negative-FH subgroups regarding age, sex, comorbid disease, MEST-C score, observation period, and therapeutic interventions. However, the eGFR value at baselines was significantly lower in the positive-FH subgroup than in the negative-FH subgroup (*P* < 0.01). On multivariate analysis, positive-FH emerged an independent determinant of poorer renal outcomes (odds ratio, 2.31; 95% confidence interval, 1.10–4.85; *P* = 0.03), after adjusting for confounding factors. eGFR at follow-up was significantly lower in the positive-FH subgroup than in the negative-FH subgroup after adjustment for age and observation period.

**Conclusions:**

Positive-FH was found in 11.6% of all IgAN patients, consistent with the incidence seen in previous literature. A significantly lower eGFR at baseline and last follow-up and unfavorable renal outcomes in the positive-FH subgroup suggest that certain genetic risk factors predisposing to renal failure may exist in a fraction of our IgAN cohort. (331 words).

**Supplementary Information:**

The online version contains supplementary material available at 10.1186/s12882-021-02425-8.

## Background

IgA nephropathy (IgAN) is the most prevalent primary glomerulonephritis worldwide [1–3]. It typically presents with persistent microscopic hematuria and proteinuria, often leading to end-stage renal disease (ESRD) over 20–30 years [1]. The diagnostic hallmark of IgAN is the predominance of IgA deposits in the mesangium with cell proliferation as well as matrix increase. IgAN is most prevalent in Asians, moderately prevalent in Europeans, and rare in those of African ancestry, suggesting ethnic and geospatial difference in the pathogenesis of IgAN [1–4]. A higher serum level of aberrantly glycosylated IgA in the hinge region, forming galactose-deficient IgA1, is found in approximately 75% of patients with IgAN and in 30–40% of their first relatives [3, 4], representing an inheritable trait. Defective IgA1 glycosylation is observed in IgAN patients who are Asians, Europeans, and African-Americans, suggesting a commonly shared mechanism among distinct ethnic groups.

IgAN is a clinically heterogeneous disease, which is caused by the interaction of multiple genes with environmental factors. A majority of IgAN cases are sporadic, while some show a familial cluster of renal diseases. Familial aggregation of IgAN is observed among IgAN patients of various ethnic backgrounds [1, 3–8], suggesting the existence of a genetic component to the disease worldwide. In most cases, the clustering pattern is autosomal dominant with variable penetrance; however, some pedigrees may fit with a more complex genetic mode [3]. The overall prevalence of positive-FH in all patients with IgAN is reported as 2 to 17% [1, 3–8], though it might be underestimated in some populations [9]. There are some controversial results regarding renal outcomes among familial IgAN patients. Studies on IgAN patients in China and Italy showed that the positive-FH subtype accounts for up to 7 and 17% of all IgAN patients, respectively, and positive-FH was consistently associated with a worse renal prognosis [5, 8]. Studies on Irish patients with IgAN reported that the positive-FH subgroup accounts for 1.8% of the total IgAN prevalence and had unfavorable outcomes [4]. In contrast, another Italian study by Izzi C et al., demonstrated that positive-FH did not worsen the renal prognosis of IgAN compared to sporadic cases [7]. Thus, the prevalence of positive-FH subtype among IgAN patients may differ by geographic area and ethnicity, and renal outcomes may vary among clinically heterogeneous subgroups.

A genetic study of familial IgAN is one of the most effective approaches to identify the major susceptibility genes and/or disease-causing genes to better understand the pathogenesis. Till date, no consistent genetic defects have been found that could predict the development or progression of IgAN. For initial assessment of the potential role of genetic factors in the pathogenesis of IgAN, we aimed to characterize the clinico-pathologic features and prognostic factors of the positive-FH subgroup of our cohort with IgAN. Generally, IgAN progresses slowly over 30 to 40 years until ESRD, although the first urinary abnormalities usually manifest at approximately 10 to 30 years of age [1, 2]. We constructed a clinical database by integrating family history, pathological findings on biopsy, therapeutic regimens, and renal function changes over the past 25 years. By following up 467 patients from an institutional IgAN registry and analyzing the corresponding data longitudinally, we showed that positive-FH of renal diseases in IgAN patients is a critical determinant of poorer renal prognosis. Moreover, the significantly lower eGFR values at renal biopsy and last visits in the positive-FH subgroup suggest that certain genetic factors predisposing to renal failure may exist in a fraction of our IgAN cohort.

## Methods

### Aim

We aimed to longitudinally observe whether a familial history of renal disease was associated with renal outcomes in IgAN patients in Japan.

### Study design

We conducted a longitudinal observation study for IgAN patients in a single center (Showa University Fujigaoka Hospital) in Japan.

### Patients

We selected histologically diagnosed IgAN patients who underwent renal biopsy between 1994 and 2019 in our institution. Patients with systemic IgA-vasculitis (Henoch-Schönlein purpura nephritis), as well as secondary forms of IgA depositions (due to liver diseases, diabetic nephropathy, cholesterol embolism, and other autoimmune diseases e.g., ulcerative colitis etc) were excluded. The possibility of C3 glomerulonephritis or unusual immune deposition diseases (e.g., immunotactoid glomerulopathy) was carefully re-evaluated by histologic review. Some uncertain cases, where the histological features of other diseases become predominant over IgAN in serial biopsies during observation, were excluded. Moreover, cases of IgAN with other coexisting predominant features (e.g., diabetic nephropathy, cholesterol embolism and microscopic polyangiitis, *etc*) were excluded. Finally, a total of 467 patients with IgAN were enrolled for the baseline study and were followed up for a mean period of 8.9 years.

The date of the renal biopsy was regarded as the baseline point for each patient. The diagnosis of IgAN was made based on inspection of renal histology including light microscopy (hematoxylin and eosin, Masson’s trichrome, periodic acid-Schiff, and methenamine silver), immunofluorescent staining (IgA, IgG, IgM, C3, C4), and electron microscopy. IgAN was histologically defined as mesangial proliferation with depositions of immunocomplexes predominantly composed of IgA and C3. For patients who underwent two or more renal biopsies, baseline data were obtained on the date of the first renal biopsy used to diagnose IgAN.

To explore the determinants of renal outcome, indicated by ESRD or > 50% reduction of renal function, we selected a subset of patients who were longitudinally followed over 2 years and an eGFR of more than 30 mL/min/1.73 m^2^ at baseline (*n* = 389; mean observation, 8.7 years, Table 1).

**Table 1:** Baseline clinical features and renal outcomes in family history-positive and -negative subgroups of IgA nephropathy

Variables	Familial History of Renal Diseases		*P* value
	Negative	Positive	
Number of Patients	*n* = 347	*n* = 42	
***Clinical backgorund at renal biopsy***			
Age (yr)	35 [15, 76]	39 [19, 70]	0.20
Age onset of hematuria and/or proteinuria (yr)	28 [5, 75]	26 [10, 66]	0.99
Gender Male (*n*)	171 (49.3)	15 (35.7)	0.10
Female (*n*)	176 (50.7)	27 (64.3)	
eGFR (ml/min/1.73m^2^)	74.00 [30.00, 153.00]	69.50 [32.00, 115.20]	**0.048**
History of Hypertension (*n*)	247 (71.2)	24 (57.1)	0.08
History of Diabetes (*n*)	17 (4.9)	2 (4.8)	1.00
Mean arterial pressure (mmHg)	85 [55, 141]	88 [67, 107]	0.46
Urinalysis of RBC (per HPF)	5 [1, 100]	10 [1, 100]	0.57
Urinary protein excretion per day (gram)	0.55 [0.00, 20.86]	0.47 [0.00, 5.05]	0.67
***Renal biopsy findings***			
C3 deposition in mesangium	1 [0, 3]	1 [0, 3]	0.34
C4 desposition in mesangium	0 [0, 3]	0 [0, 1]	0.24
IgA deposition in mesangium	2 [1, 3]	2 [1, 3]	0.70
IgG deposition along the capillary loops	0 [0, 3]	0 [0, 2]	0.55
**Oxford MEST-C Score**			
M Score 1 (proportion, %)	169 (48.7)	21 (50.0)	1.00
E Score 1 (proportion, %)	34 (9.8)	6 (14.3)	0.42
S Score 1 (proportion, %)	57 (16.4)	5 (11.9)	0.66
T Score (0–2) by 1 increase	0.27 (0.50)	0.31 (0.47)	0.66
C score 1 to 0 (proportion, %)	66 (19.0)	6 (19.0)	1.00
GBM thickness (nm)	293.3 [131,422]	294.0 [159,455]	0.93
Number of patients with thin GBM(%)	13 (3.7)	2 (4.8)	0.69
***Renal Outcomes***			
Age at last evaluation (yr)	46.00 [18.00, 87.00]	45.00 [24.00, 80.00]	0.44
Observational peroid (yr)	9.00 [2.00, 25.00]	8.00 [2.00, 25.00]	0.47
eGFR (ml/min/1.73 m^2^)	66.00 [5.00, 160.50]	54.00 [5.00, 106.50]	**0.02**
Mean eGFR reduction (ml/min/1.73m^2^/year)	−0.88 [−24.00, 10.23]	−1.09 [−13.20, 5.25]	0.35
More than 50% reduction of eGFR(proportion, %)	41 (11.8)	10 (23.8)	**0.048**
CKD stage 5 (proportion, %)	27 (7.8)	8 (19.0)	**0.04**
CKD stage 4 or 5 (proportion, %)	42 (12.1)	12 (28.6)	**0.01**
Primary outcome (proportion, %)	41 (11.8)	10 (23.8)	**0.048**
***Therapy and intervension***			
Administration of immunosupressant (proportion %)	6 (1.7)	1 (2.4)	0.55
Adminstration of RAS inhibitors (%)	218 (62.8)	31 (73.8)	0.18
Tonsillectomy plus steroid-pulse therapy (%)	171 (49.3)	25 (59.5)	0.25
long-term oral prednisolone (%)	35 (10.1)	2 (4.8)	0.40

A total 389 individuals for whom longitudinal progression data are available for more than 2 years (mean follow-up time 8.7 years) are studied. Values for categorial variables are given as number or (percentage) or [range]. MEST-C scores are determined according to the Oxford Classification [10]. Endocapillary hypercellularity: absent (E0) or present (E1); Segmental glomerulosclerosis absent (S0) or present (S1); Tubular atrophy/interstitial fibrosis #25% (T0), 26–50% (T1), or > 50% (T2); Cellular/fibro-cellular crescents absent (C0), present in at least one glomerulus (C1), in > 25% of glomeruli (C2). Mesangial depositions of IgA, C3, C4, as well as capillary IgG depositions are examined by immunofluorescence staining and are scored as four subclasses: 0 (absent), 1 (weak), 2 (Intermediate), and 3 (strong). Thickness of glomerular basement membrane is measured for several different segments along the capillaries on electron microscopy images and average thickness less than 200 nm is judged as thin glomerular basement membrane (TGBM)

The primary outcome consists of a hard endpoint (ESRD eGFR< 15 mL/min/1.73 m^2^) and a surrogate endpoint (50% or more reduction in renal function) [11]. eGFR, estimated glomerular filtration rate; FH, familial history; MAP, mean arterial pressure; RAS, renin angiotensin system. Quantitative variables are expressed as absolute number (mean with [range]), or frequency (%). Chi-square and t-tests are used for comparison. Results that have *P* < 0.05 are indicated in *bold*

### Clinical, laboratory, and pathological evaluation

Baseline data at the time of the renal biopsy were extracted from medical records and interviews. Clinical data, including age, sex, history of diabetes and/or hypertension, renal familial history, urinary protein excretion levels, urinalysis results, mean arterial pressure (MAP), and serum creatinine values were collected. A positive history of hypertension was assigned if the patient had a systolic blood pressure > 130 mmHg at the initial renal biopsy or previously/concurrently used anti-hypertension drugs.

Additionally, data regarding the renal familial history; therapeutic interventions, including the use of renin-angiotensin-aldosterone system (RAS) inhibitors, tonsillectomy with subsequent steroid-pulse therapy, oral corticosteroid treatment, immunosuppressive therapy; and current renal function on the last clinical visits (as of February 2020) were obtained from our hospital records. Tonsillectomy with subsequent steroid-pulse therapy regimens was referred to the Hotta’s protocol [12]. For patients aged > 20 years, the eGFR was calculated using a standard formula [13]. For patients aged between 15 and 19 years, the eGFR was calculated using the Cockcroft & Gault’s formula and multiplied by 0.764 [14]. Two or more investigators blinded to the outpatient’s status collected all the clinical data. ESRD was defined as individuals who underwent renal transplantation or dialysis or had diminished eGFR (less than 5 mL/min/1.73 m^2^).

Clinical histological grading of renal biopsy specimens was determined by the Oxford classification (i.e., Oxford MEST-C scores) [10]. Renal histology was independently reviewed by two or more nephrologists in a blinded manner. Additionally, the thickness of the glomerular basement membrane (GBM) was evaluated by either digital-image analysis (CellSens computer software, Olympus, Tokyo, Japan) or manual measurement of non-digital photographs by 10X eyepiece magnifier. An average GBM thickness was calculated for at least three different capillary segments and values below 200 nm were defined as thin GBM.

### Definition of positive familial history in patients with IgAN

We defined a subset of positive-FH by subclassifying IgAN patients under the categories of either ‘familial’ or ‘suspected-familial’ renal history, employing the definitions that fit with those of previous literature [3–5]: specifically, “familial” renal history is ascertained when at least two first-degree family members have a biopsy-proven IgAN; and “suspected-familial,” is assigned, if one first-degree family member has a biopsy-proven IgAN and other relatives have persistent microscopic hematuria and/or renal failure with chronic kidney disease of unknown cause. The family history was updated based on the latest medical record.

### Primary renal outcomes

The primary renal outcome was a composite of a hard endpoint, i.e. ESRD (eGFR < 15 mL/min/1.73 m^2^) and surrogate endpoint (a 50% or more reduction in the eGFR from baseline value), consistent with those of previous reports [11]. The observation was terminated once a patient started dialysis or underwent renal transplantation.

### Statistical analysis

For continuous variables, differences between the two groups were evaluated using the unpaired t-test or Mann–Whitney U test, as appropriate. Differences in the proportions of different patient groups were evaluated by the Fisher’s extract test. A *P*-value < 0.05 was considered statistically significant. Univariate renal survival comparisons were performed using the log-rank scale test. All univariate tests were two-sided with a significance level of 0.05.

To estimate the adjusted relative risk of FH for the composite endpoints (eGFR< 15 mL/min/1.73 m^2^ and > 50% reduction in renal function from baseline), Cox proportional hazards analysis was conducted for a subset of patients who underwent longer observation (> 2 years) and maintained eGFR> 30 mL/min/1.73 m^2^. To avoid multicollinearity and overfitting, we carefully selected several explanatory variables for inclusion in a multivariate analysis based on the previous studies [11, 15]. Given the slow progressive nature of IgAN, which takes 20 to 30 years from onset to ESRD, age and observation period may affect the endpoint eGFR. Analysis of co-variance (ANCOVA) was used to compare mean differences in renal function (eGFR) on last evaluation between the positive- and negative-FH subgroups. A one-way ANCOVA was employed with age or observation period as the covariate, FH as the independent variable, and current eGFR values on last evaluation as the dependent variable. Normality was checked using Levene’s test. Statistical analyses were performed using R version 3.6.1 statistical software (The R foundation for statistical computing, Vienna, Austria).

## Results

### Patient selection and baseline clinical characteristics

**Fig. 1:**
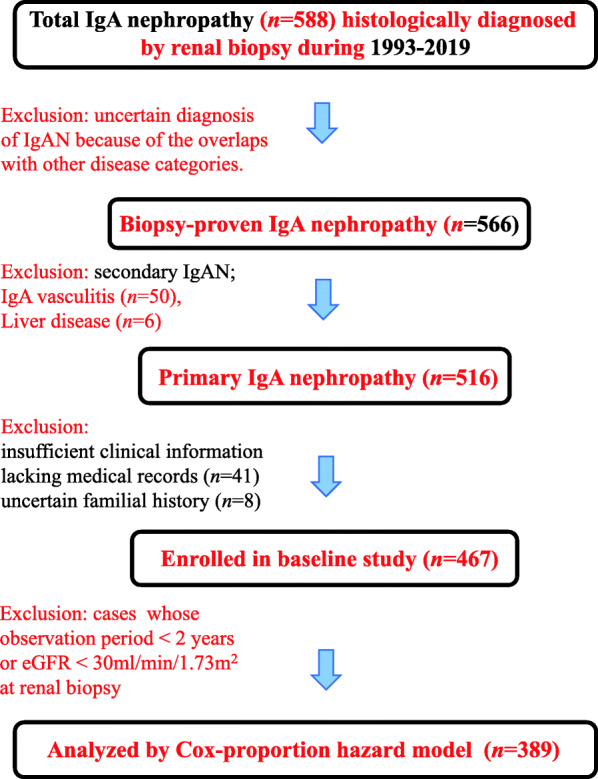
Flowchart of selections patients enrolled in the present study. The data of 389 individuals with IgA nephropathy who were followed-up for more than 2 years (mean follow-up time 8.7 years) and had an eGFR > 30 mL/min/1.73 m^2^ are analyzed using the Cox regression analysis to determine the predictors of ESRD

From a total of 516 participants (Fig. 1 and Additional file 1), we selected 467 IgAN patients who underwent clinical follow-up for more than 2 years and maintained renal function eGFR more than 30 mL/min/1.73 m^2^ at initial diagnosis (Additional file 1). Among these enrolled subjects, 54 patients had a positive FH (11.6%), of which 36 patients (66%) were classified to have a “definite” FH and 18 (34%) were classified to have “suspected” FH of renal disease. With regards to the transmission pattern, 47 patients had a multi-generational clustering of disease, while five showed a single-generation pedigree including only affected siblings with healthy parents. At the time of initial diagnosis by renal biopsy, there were no inter-group differences in sex and age. Moreover, the severity of urinary protein excretion, histological grade, and comorbidity of hypertension and diabetes mellitus did not differ between the FH-positive and negative-FH subgroups. Notably, the initial eGFR at renal biopsy was remarkably lower in patients with positive-FH than in those with negative-FH (65 vs 73 mL/min/1.73 m^2^, respectively, *P* = 0.01).

We next evaluated the histological grade of biopsy specimens according to the Oxford classification. The intensity of mesangial depositions of C3, C4, IgA, and IgG depositions along the capillary loops did not differ between the two groups. In addition, based on the Oxford MEST-C scores, the pathological severity did not differ between the two subgroups. On electron microscopy, average GBM thickness did not significantly differ between two subgroups, nor did the frequency in cases of thin GBM (< 200 nm).

### Treatment interventions, follow-up period, and primary outcomes

**Fig. 2:**
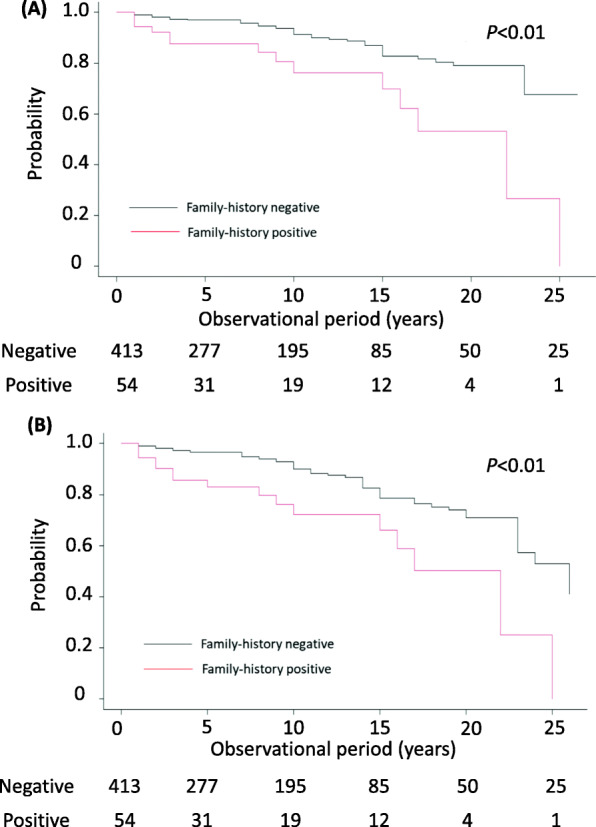
Comparison of renal survival between IgAN patients of positive- and negative- family history subgroups. Renal survival is analyzed using the log- rank scale test and compared between the two subgroups of IgAN patients, i.e., those with a positive- or negative-FH. **A** A hard endpoint of ESRD (eGFR less than 15 mL/min/1.73 m^2^) was used. **B** The combined renal outcomes, consisting of both the hard endpoint (eGFR< 15 mL/min/1.73 m^2^) and surrogate endpoint (a 50% or more reduction in renal function), were used. IgAN, IgA nephropathy; FH, familial history of renal diseases; ESRD, end-stage renal disease; eGFR, estimated glomerular filtration rate

We evaluated differences in therapeutic approaches with regards to tonsillectomy with steroid-pulse therapy, use of long-term oral steroids, RAS inhibitors, and immunosuppressant. There were no differences in the therapeutic regimens between the two subgroups. The duration of the follow-up period was almost the same in the two subgroups (9.0 years vs 8.0 years, negative-FH vs positive-FH). With regards to the composite renal outcome, the positive-FH subgroup clearly showed a worse renal prognosis than the negative-FH subgroup (*P* < 0.01) (Fig. 2A and B). Additionally, the decline in the eGFR per year was steeper in patients with positive-FH than in those with negative-FH (*P* = 0.01) (Additional file 1).

### Univariate and multivariate analysis of variables contributing to primary outcomes

The predictors of ESRD were estimated by univariate and multivariate Cox proportional hazard analyses for the subset of IgAN patients (*n* = 389) who underwent longer follow-up (> 2 years) and had an eGFR > 30 mL/min/1.73 m^2^ (Table 2). In the univariate analysis, age, history of hypertension, proteinuria, MAP, eGFR, positive-FH, Oxford scores (M-, and T-), use of RAS inhibitors, and combined tonsillectomy with steroid therapy were associated with the primary renal outcomes. We also performed a multivariate Cox proportional hazards analysis with selected prognostic variables (positive-FH, age, MAP, eGFR, proteinuria, and Oxford classification T-scores) (Table 3). In the multivariate analysis, positive-FH, as well as eGFR and urinary protein excretion, were independent determinants of renal outcomes (odds ratio, 2.31; 95% confidence interval, 1.10–4.85; *P* = 0.03).

**Table 2:** Univariable Cox proportional hazards analysis for IgAN patients

Variables	Odds ratio	95% confidence Interval		*P* value
***Clinical background at Renal Biopsy***				
History of Hypertension (presence or absence)	3.07	1.75	5.39	**< 0.001**
Mean arterial pressure (1 mmHg increase)	1.02	1.00	1.04	**0.02**
Age at renal biopsy (1 increase)	1.03	1.01	1.05	**0.03**
eGFR (ml/min/1.73 m^2^)	0.97	0.95	0.98	**< 0.001**
Sex, Male	1.08	0.63	1.88	0.78
Urinary sediment RBC (per HPF) by 1 increase	1.01	0.98	1.03	0.62
Urinary protein excretion per day (g/day) by 1-unit increase	1.18	1.08	1.30	**< 0.001**
Familial History of renal disease (positive or negative)	2.40	1.19	4.83	**0.02**
Diabetes mellitus (presence or absence)	1.32	0.47	3.72	0.59
***Renal Histology***				
**MEST-C score**				
M score 1 to 0	1.84	1.05	3.20	**0.03**
E score 1 to 0	1.47	0.86	3.15	0.33
S score 1 to 0	1.88	0.96	3.70	0.06
T score by 1 increase	1.86	1.19	2.90	**0.01**
C Score 1 to 0	1.59	0.81	3.10	0.18
**Immune depostions**				
IgA deposition in mesangium	1.10	0.70	1.72	0.70
C3 deposition in mesangium	1.43	0.92	2.21	0.11
C4 deposition in mesanigum	0.61	0.32	1.18	0.14
IgG deposition along capillary loop	1.49	0.96	2.34	0.08
**GBM Thickness**	0.10	0.99	1.00	0.57
***Therapeutic Intervention***				
Tonsillectomy with steroid-pulse therapy	0.51	0.26	1.00	0.05
Administration of Immuno-suppresants	< 0.001	0.00	infinity	1.00
Long-term prednisolone use	2.12	1.14	3.95	**0.02**
Use of RAS blockades	4.55	1.09	19.1	**0.04**

The odds ratio and 95% confidence intervals are estimated using multivariable Cox proportional hazards analysis in IgA nephropathy patients (*n* = 389) who were followed up for more than two years and had a baselines eGFR > 30 mL/min/1.73 m^2^. The MEST-C scores are determined according to the Oxford Classification [10]. Endocapillary hypercellularity: absent (E0) or present (E1); Segmental glomerulosclerosis absent (S0) or present (S1); Tubular atrophy/interstitial fibrosis #25% (T0), 26–50% (T1), or > 50% (T2); Cellular/fibro-cellular crescents absent (C0), present in at least one glomerulus (C1), in > 25% of glomeruli (C2). Mesangial depositions of IgA, C3, C4, as well as capillary IgG depositions are examined using immunofluorescence staining and are graded into four subclasses: 0 (absent), 1 (weak), 2 (Intermediate), and 3 (strong). Thickness of glomerular basement membrane is measured for several different segments along the capillaries on electron microscopy images and average thickness less than 200 nm is judged as thin glomerular basement membrane (TGBM). eGFR, estimated glomerular filtration rate; FH, familial history; MAP, mean arterial pressure; RAS, renin angiotensin system. Results that have *P* < 0.05 are indicated in *bold*

**Table 3:** Multivariable Cox proportional hazards analysis for IgAN patients

Factors	Odds ratio	95% confidence Interval		*P* value
***Clinical background at Renal Biopsy***				
History of Hypertension (presence to absence)	1.39	0.68	2.85	0.37
Mean arterial pressure (1 mmHg increase)	1	0.98	1.02	0.76
Age at renal biopsy (1 increase)	1.01	0.98	1.03	0.57
eGFR (ml/min/1.73m^2^)	0.97	0.96	0.99	**< 0.01**
Urinary protein (g/day) by 1 increase	1.17	1.04	1.31	**0.01**
Familial History (Positive or Negative)	2.31	1.1	4.85	**0.03**
Renal biopsy findings				
MEST-C T score by 1 increase	1	0.57	1.73	0.98
***Treatment Intervention***				
Long-term prednisolone use	1.85	0.94	3.64	0.08
Use of RAS inhibitors	2.65	0.61	11.5	0.19

The odds ratio and 95% confidence intervals are estimated using multivariable Cox proportional hazards analysis in IgA nephropathy patients (*n* = 389) who were followed up for more than two years. MEST-C scores are determined according to the Oxford classification [10]. Cellular/fibro-cellular crescents are scored as absent (C0), present in at least one glomerulus (C1), or present in > 25% of glomeruli (C2). eGFR, estimated glomerular filtration rate; RAS, renin angiotensin system; FH, familial history [of renal disease]. *P* values < 0.05 are boldfaced

### Comparison of current eGFR between FH-positive and -negative subgroups by ANCOVA

**Fig. 3:**
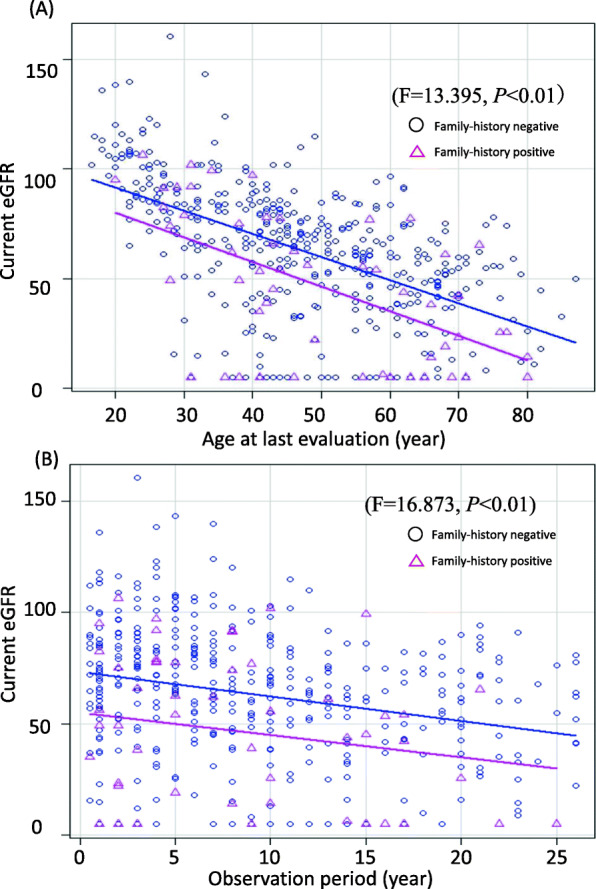
Difference in current eGFR values adjusted for age and observation period between positive- and negative-FH subgroups of IgAN. eGFR values measured on the last clinical visitare compared between the positive- and negative- FH subgroups using a one-way ANCOVA, adjusting for age (**A**) or observation period (**B**). Lines indicate the mean of the last available eGFR values in each subgroup

Current eGFR determined at the follow-up visit was remarkably lower in patients with positive-FH than in those with negative-FH (54.0 vs 66.0 mL/min/1.73 m^2^, respectively, *P* = 0.02). To investigate the possible effect of confounding factors such as age and observation period on the endpoint eGFR, we performed a one-way ANCOVA to compare mean differences in the last available eGFR values between the positive- and negative-FH subgroups (Fig. 3A and B). ANCOVA, with age as the covariate, revealed that renal function was significantly lower in the positive-FH subgroup than in the negative-FH subgroup (F = 13.395, *P* < 0.01) (Fig. 3A). Moreover, mean eGFR levels were significantly lower in the positive-FH subgroup than in the negative-FH subgroup even after adjusting for the observational period (F = 16.873, *P* < 0.01) (Fig. 3B).

## Discussion

The present study in a single-center cohort revealed that IgAN patients with a FH of any kidney disease have an increased risk of ESRD than those without any such FH. The single-center study design allowed us to minimize the ascertainment biases arising from histological diagnosis and longitudinal follow-up of patients over an average of 8.9 years. Our observation indicates that the prevalence of positive-FH was 11.6% among histologically proven, primary IgAN cases. The incidence of family clustering is almost comparable to that in previous studies: 6.7% in China [8] and 17.2–34% in Italy [5] (Additional file 2). Other studies with Caucasians showed a lower frequency of familial aggregation in total sporadic cases: 1.8% in Ireland [6] and 1.4% in the US [15]. The difference in frequency of familial clustering may be due to the geographic and/or racial difference in genetic background. Moreover, biopsy policy and diagnostic criteria for familial aggregation of IgAN may vary among the countries.

Our study indicates that the IgAN subgroup with positive-FH had an unfavorable renal outcome compared with the negative-FH subgroup. The results were in good agreement with those of two previous studies for European and Chinese patients with familial IgAN [8]. A prior nationwide study in Japanese, primarily sporadic IgAN patients (*n* = 2283) showed that approximately 5% of all individuals with IgAN had FH of chronic glomerulonephritis; the incidence was comparable to previous studies [16]. An FH of renal failure or chronic glomerulonephritis was associated with a poorer renal prognosis with hazard ratios of 2.49 (*P* = 0.015) and 2.07 (*P* = 0.031), respectively. However, the etiologies of chronic glomerulonephritis remained unclear. A recent cohort study in China with familial and sporadic IgAN cases showed that the familial cases developed ESRD 5 years earlier than the sporadic cases did [8]. However, discordant observation was reported in a Spanish study reported by Izzi C et al. [7], showing no differences in renal outcome between familial and sporadic cases. Several possibilities responsible for the contradictory observations should be taken into consideration. First, there might be potential heterogeneity of genetic mechanisms underlying familial IgAN. IgAN is a heterogeneous disorder and subclinical inherited diseases might underlie or modify the clinical picture of IgAN, thereby affecting the renal prognosis [17]. Secondly, clinical assignment of familial IgAN may vary among researchers. A previous study by Izzi C et al. adopted a strict definition for positive-FH: patients with at least two first-degree family members who had biopsy-proven IgAN were classified into the “familial” IgAN subgroup [7]. The diagnosis of “suspected IgAN” in our cohort is not histologically proven and may include coincidentally other types of genetic kidney disorders. Of note, we could not find any difference in the age of onset and MEST-C score at initial biopsy. However, familial cases were diagnosed younger and showed histologically milder disease at an earlier stage, pointing to the importance of earlier diagnosis [5, 8, 15]. More attention for familial clustering and active screening of family members for eGFR and urinalysis are recommended in clinical practice.

Our Cox regression analysis demonstrated that the positive-FH, together with proteinuria, and low eGFR, are independent determinants that impact renal outcome, as seen in previous studies [2, 11, 16, 18]. At the initial point of histological diagnosis of IgAN, patients in the positive-FH subgroup presented a significantly lower initial eGFR. However, there was no difference seen with co-occurrence of hypertension and/or proteinuria, both of which are reported to be critical prognostic factors for renal outcomes [2, 11, 16, 18]. Moreover, current eGFR values were also lower in the positive-FH subgroup than in the negative-FH subgroup, even after adjustment for age and observation period. These findings suggest that a prior decrease in eGFR might genetically predispose and contribute to the renal prognosis of FH-positive IgAN patients. It is tempting to speculate that familial IgAN represents a distinct subset of IgAN, which progresses faster into ESRD. In other words, IgAN individuals in positive-FH subgroup harbor as-yet undefined genetic factors that accelerate a fall in eGFR over a period of more than 10 years. A recent study demonstrated that some properties in kidney development, *i.e*, the nephron number and immune susceptibility against pathogen, varies across ethnicities, reflecting an innately equipped genetic background [1, 3, 4]. Further study is necessary to elucidate the genetic factors involved in our cohort of IgAN patients.

Numerous studies have recognized familial aggregation of IgAN. However, the genetic basis underlying familial disease remains largely unclear. Both geo-ethnic variation in the prevalence of IgAN and familial clustering of the disease support the notion that genetic factors may contribute to the pathogenesis of IgAN. Genome wide association studies (GWAS) have identified many common risk loci in sporadic IgAN cases. More than 20 distinct genome-wide significant loci of small to moderate effects reported to date could account for 6–8% of the disease risk [3, 4]. IgAN may be caused by multiple hits of several candidate genes involved in antigen presentation, gut mucosal immunity, IgA biology, and complement pathway [19]. To date, no single causal genes have yet been identified in familial IgA cases, suggesting that the disease is multifactorial or has a complex trait, where one or more genes are implicated in combination with environmental factors [3, 4, 19]. A considerable portion of missing heritability so far reported in GWAS could be explained by multiple rare variants [17, 20–22]. Recent studies with exome analysis revealed certain familial or sporadic IgAN harbor variants in known renal disease-causing genes, suggesting that IgAN may be driven or modified by genes of other familial renal disorders [17, 20–22]. Thus, next generation sequencing will help improve our understanding of genes implicated in the pathogenesis of IgAN.

Our study has several limitations. It remains uncertain whether our observations are generalizable because they were based on the data from a single center. Firstly, the number of endpoint events was not sufficient to accommodate all suitable predictor variables. This problem may be ascribed to the limited observation period in our study (average, 8.9 years), considering that IgAN progresses slowly over 20–30 years until ESRD [1, 2]. IgAN in East Asian countries, including Japan, may have relatively more favorable outcomes than in European countries. Thus, a longer observation with more participant numbers is warranted. Secondly, our screening for family members might be inadequate and underdiagnosed: we could not examine the urinalysis data for all relatives of patients with positive FH. Several cases were enrolled into the positive-FH subgroups based on urinalysis (i.e., persistent hematuria); cases of non-glomerular hematuria, for example, nutcracker syndrome or ureterolith, were excluded. Thirdly, the definition of relevant FH in familial IgAN may vary among clinicians. Using the strict criteria defined for our cohort, we could only analyze 54 patients with a positive FH. The paucity of the number of subjects would not only lead to a lack of statistical analytic power but would also underestimate the relevance of other prognostic factors (proteinuria, and hypertension, *etc*).

## Conclusions

Herein, we showed that a positive-FH subset accounted for approximately 11.6% of all patients with IgAN in our cohort. The prevalence of familial clustering is comparable to that previously reported among East Asians. Our results corroborate those of previous studies from China and Italy, indicating that the renal outcomes are worse in patients with positive-FH than in those with negative-FH. The assessment for familial clustering and eGFR measurement would be important to predict ESRD risk, particularly in IgAN patients for whom renal biopsy is difficult to perform. Further studies with more patients are needed to clarify the contribution of genetic factors in the pathogenesis of IgAN.

## Supplementary Information

**Additional file 1. **Baseline clinical and histological features of IgA nephropathy patients initially enrolled in this study (*n* = 467).

**Additional file 2.** Summary of studies reporting clinical features and renal prognosis of familial IgA nephropathy.

## Data Availability

Not applicable.

## Abbreviations

IgAN: IgA nephropathy
eGFR: Estimated glomerular filtration rate
ESRD: End-stage renal disease
FH: Familial history [of renal diseases]
MAP: Mean arterial pressure
RAS: Renin angiotensin system
GBM: Glomerular basement membrane
